# Calcination Improves the In Vivo Efficacy of a Montmorillonite Clay to Bind Aflatoxin G1 in Broiler Chickens: A Toxicokinetic Approach

**DOI:** 10.3390/toxins12100660

**Published:** 2020-10-18

**Authors:** Roua Rejeb, Siegrid De Baere, Mathias Devreese, Richard Ducatelle, Siska Croubels, Madiha Hadj Ayed, Achraf Ghorbal, Gunther Antonissen

**Affiliations:** 1 LR18AG01, ISA-CM-BP, 47, Institut Supérieur Agronomique de Chott-Mariem, Université de Sousse, 4042 Sousse, Tunisia; mediha.ayed@yahoo.fr; 2 Department of Pathology, Bacteriology and Avian Diseases, Faculty of Veterinary Medicine, Ghent University, 9820 Merelbeke, Belgium; Richard.Ducatelle@UGent.be; 3 Department of Pharmacology, Toxicology and Biochemistry, Faculty of Veterinary Medicine, Ghent University, 9820 Merelbeke, Belgium; Siegrid.DeBaere@UGent.be (S.D.B.); Mathias.Devreese@UGent.be (M.D.); Siska.Croubels@UGent.be (S.C.); 4 Research Laboratory LR18ES33, National Engineering School of Gabes, University of Gabes, Avenue Omar Ibn El Khattab, 6029 Gabes, Tunisia; achraf.ghorbal.issat@gmail.com

**Keywords:** AFG1, toxicokinetics, LC-MS/MS, LC-HRMS, calcination, montmorillonite clay, broiler chicken

## Abstract

The goal of this study was to investigate the toxicokinetic characteristics of aflatoxin G1 (AFG1) in broiler chickens and the effect of calcination of a Tunisian montmorillonite clay on the in vivo absorption of AFG1. In this study, broiler chickens were randomly distributed into four groups of 10 animals. Group 1 was administered AFG1 (2 mg/kg body weight (BW)) by single intravenous injection (IV), group 2 received an intra-crop bolus (PO) of AFG1 without any clay, group 3 was dosed AFG1 PO together with an oral bolus of purified clay (CP), and group 4 received AFG1 PO with an oral bolus of calcined clay. A significant difference in the area under the curve (AUC_0-t_) was observed for group 4 (6.78 ± 4.24 h*ng/mL) in comparison with group 2 (12.83 ± 4.19 h*ng/mL). A significant reduction of the oral bioavailability of AFG1 was observed for group 4 (7.61 ± 4.76%) compared with group 2 (14.40 ± 4.70%), while no significant effect was observed of CP. In this experiment, no phase I nor phase II metabolites of AFG1 were observed. These findings confirm that calcination of the purified montmorillonite clay enhances the adsorption of AFG1 in the gastrointestinal tract after oral administration, thereby reducing its bioavailability, thus reducing its toxic effects.

## 1. Introduction

Mycotoxins are secondary fungal metabolites present on a multitude of crops. Incorrect storage conditions, stress conditions during pollination, warm ambient temperatures and drought conditions during the growing season, and insect damage can lead to aflatoxins (AFs) contamination of food and feed commodities [1]. Aflatoxins are difuranocoumarin derivatives produced mainly by *Aspergillus flavus* and *Aspergillus parasiticus* strains and can contaminate many different crops, particularly maize, groundnuts, and wheat [2,3,4]. Aflatoxin contamination of feed and animal products can differ depending on geographical location, country development level, and climatic conditions. Aflatoxin production mainly occurs in regions with tropical or subtropical climates [5]. Among the various types of AFs, which include aflatoxin B1 (AFB1), aflatoxin B2 (AFB2), aflatoxin G1 (AFG1), and aflatoxin G2 (AFG2), AFG1 is the second most toxic aflatoxin after AFB_1_ [6,7,8]. Moreover, the International Agency for Research on Cancer has classified AFG1 as a group 2B toxin, stating that this may be carcinogenic to humans [9]. Aflatoxins form colorless to pale-yellow crystals. These crystals are intensely fluorescent in ultraviolet light, emitting blue (AFB1 and AFB2) or green (AFG1) and green-blue (AFG2) fluorescence [10]. In poultry, the effects of aflatoxins include a.o. hepatotoxicity, impaired productivity, decreased egg production, inferior eggshell quality, lower carcass quality, and increased susceptibility to other diseases [11]. Aflatoxicoses reduce the performance of broiler chickens due to decreased feed intake, increased feed conversion, reduced weight gain [12], and altered visceral organ weights [13,14,15]. Feeding a high dose (3.5 mg AFs/kg feed) of an AFs mixture (79% AFB1, 16% AFG1, 4% AFB2, and 1% AFG1) to broilers significantly reduced their body weight and increased their liver and kidney weights [16]. Moreover, at low chronic doses of AFB1 (0.03 mg/kg of feed), a reduction of body weight gain of 30% has been observed [17]. Oral administration of AFG1 can induce hyperplastic lesions and adenocarcinoma of the lung in NIH mice [18]. AFG1 also caused the development of liver tumors in experimental animals but generally at a lower incidence than AFB1 [19]. Ma et al. [20] studied the characteristics of the interaction between AFB1/AFG1 and calf thymus DNA in a pH 7.4 Tris-HCl buffer. The results demonstrated that both AFB1 and AFG1 bound to calf thymus DNA, forming complexes through hydrogen bonding. 

To protect poultry and other livestock from the deleterious effects of these toxins, various post-harvest intervention strategies have been developed. One such approach is adding mycotoxin detoxifying agents to the feed [21,22,23]. These detoxifiers can be divided into two different classes, namely mycotoxin binders and mycotoxin modifiers. Mycotoxin binders are able to bind mycotoxins, reducing their oral bioavailability by the formation of a non-resorbable binder–toxin complex in the intestinal tract that is eliminated through the feces [24,25]. The adsorption processes between mycotoxin and adsorbing agents are interactions between the surface of the adsorbent (e.g., mycotoxin binder) and the adsorbate (e.g., toxin). The adsorption capacity of clay minerals depends on their physico-chemical properties [22]. Adsorption to clays is not limited to the surface of the clay particles but extends also to the interlayer spaces of the clay. These spaces can increase when the clay swells, thereby increasing the number of binding sites [26]. Among the mycotoxin binders, phyllosilicate clays are the largest group and have been used in numerous research trials [27,28,29]. Different studies demonstrated that mineral adsorbents including bentonite, zeolite, montmorillonite, and hydrated sodium calcium aluminosilicate can bind or adsorb mycotoxins to their interlayer spaces, external surface, and edges [21,30,31,32]. Montmorillonite, an aluminum silicate, is characterized by a permanent negatively charged surface and exchangeable cations in the interlayer space. Montmorillonite clays have excellent effectiveness in binding polar mycotoxins such as aflatoxins, and consequently reducing their toxicity [33,34,35]. The physico-chemical properties of clay minerals can be affected by various treatments, including thermal treatment, acid activation, pillaring, organic modification with polymers, or cation and anion exchange [36,37,38]. These modified clays might bind some of the mycotoxins better than the untreated clay [39,40]. Recently, it has been observed that calcination of purified Tunisian montmorillonite clay (CC) at 550 °C enhanced the in vitro adsorption efficacy for AFB2, AFG1, AFG2, and zearalenone (ZEN) compared to the non-heat-treated purified form (CP) of the same clay. The binding capacity of AFB1 was almost 100% for both purified and calcined clay [38]. Calcination is a process in which clay minerals are heated to different temperatures. In vitro studies are used as a screening tool for the potential of substances to act as mycotoxin binder. However, in vitro studies do not adequately mimic the conditions in the digestive tract, the differences between target animals and their metabolism, and, therefore, cannot be used to demonstrate efficacy under practical conditions. Some in vivo experiments are thus necessary for their assessment [41]. The in vivo efficacy of a mycotoxin detoxifier can be evaluated by measuring the impact on unspecific parameters such as animal performance, histological changes, and hematological parameters [42,43,44]. Nevertheless, the European Food Safety Authority (EFSA) asserts that toxicokinetic studies have to be performed in order to evaluate the oral bioavailability and the absorption/excretion of mycotoxins mixed with the binder [41]. According to the EFSA, one of the most relevant parameters for evaluating the effectiveness of these products against mycotoxins is the plasma concentration of these toxins or their main metabolites or interaction products with macromolecules such as nucleic acids or proteins [45].

To estimate the health risk in affected animals and also to evaluate the possible carry-over of AFs into tissues and products derived from animals, the knowledge of the adsorption, distribution, metabolism, and excretion (ADME) process of these mycotoxins is fundamental. The results of an in situ perfusion technique employed on rats demonstrated that absorption of AFs in the small intestine is a very fast process that pursues first-order kinetics, with an absorption rate constant (k_a_) of 5.84 ± 0.05, 4.06 ± 0.09, 2.09 ± 0.03, and 1.58 ± 0.04 h^−1^, respectively, and with absorption half-lives of 7.12 (AFB1), 10.24 (AFB2), 19.90 (AFG1), and 26.30 (AFG2) min [46]. Similarly, following oral administration of AFB1 (0.25 mg/kg BW) to rats, the plasma concentration indicates a rapid absorption, with a maximum concentration at 10 min [47]. After intravenous injection (IV) administration of ^14^C-labelled AFB1 in mice, rats, and monkeys, the excretion of the toxin is high during the initial 24 h following single intravenous administration. However, the total recovery of the administered AFB1 is between 72% and 80% during the first 100 h after the IV injection [48]. After oral administration of ^14^C-labelled AFB1 to laying hens, 71% might be recovered within 7 days post-administration. In the same study, only 28% of the administered dose of AFB1 could be recovered during the first 24 h [49]. In this regard, Hussain et al. [50] demonstrated that feeding of three levels of AFB1 (1.6, 3.2, and 6.4 mg/kg BW) to broiler chicks of three different ages (7, 14, and 28 days) for 7 days results in a slow residues clearance after the withdrawal of AF-contaminated feed. AFB1 is detectable in the liver and muscles for a long period when the birds are younger and when they are fed high AFB1 dietary levels. This suggests that birds develop a more sufficient mechanism of metabolizing AFB1 with increasing age. 

To the best of our knowledge, no in vivo toxicokinetic studies of AFG1 with or without mycotoxin detoxifier in broiler chickens have been published, and only one study explored the effect of calcination on mycotoxin binding [31]. The aim of this study was to investigate a) the toxicokinetic parameters of AFG1 and b) the effect of calcination of purified montmorillonite clay collected from Jebel Aïdoudi (El Hamma, Gabes, Tunisia) on different toxicokinetic parameters of AFG1 in broiler chickens. First, a UHPLC-MS/MS method for the quantitative determination of AFG1 in broiler chicken plasma was optimized and validated. Second, a toxicokinetic study with AFG1 was done in broiler chickens. Finally, the LC-MS/MS method was transferred to a ultra-high-performance liquid chromatography-high-resolution mass spectrometry (UHPLC-HRMS) instrument, and plasma samples were analyzed to evaluate the presence of possible phase I and phase II metabolites of AFG1. 

## 2. Results

### 2.1. UHPLC-MS/MS Plasma AFG1 Method Validation

The in-house UHPLC-MS/MS method was sensitive for AFG1. For the calibration model, a linear, 1/x^2^ weighed, fitting was applied. The results for linearity (r and g) and sensitivity (limit of quantitation (LOQ) and limit of detection (LOD)) were summarized in Table 1. Linear matrix-matched calibration curves were reached (range of 0.50–200 ng/mL) with r = 0.9987 ± 0.0008 (*n* = 3) and g = 4.50 ± 1.48% (*n* = 3) values falling within the limits of ≥0.99 and ≤20%, respectively. The LOQ was set at 0.50 ng/mL, whereas the LOD was found to be 0.16 ng/mL. Furthermore, the within-day and between-day precision and accuracy fell within the acceptance criteria specified in the guideline VICH GL49 [51], and are given in Supplementary Table S2. Moreover, the reconstitution solvent was injected after the highest calibrator sample in each analytical batch. In some, but not all, solvent samples, a small peak at the elution zone of AFG1 was observed, indicating a mean carry-over of 0.06% on the UHPLC-MS/MS instrument. Moreover, the response of the eventual peak at the elution zone of AFG1 fell below 20% of that of the corresponding peak in the LOQ samples. The specificity of the method was evaluated by the analysis of a blank plasma sample in each analytical batch. In some blank plasma samples, a small peak at the elution zone of AFG1 was observed, but the response was below 20% of that of the LOQ value (0.5 ng/mL).

In Figure S1, UHPLC-MS/MS chromatograms are shown of the analysis of a blank sample (A), a blank sample spiked at the LOQ level of 0.5 ng/mL (B), and two real samples that were taken 10 min after the IV (C) and 30 min after the intra-crop bolus (PO) (D) of 2 mg/kg BW of AFG1 to two chickens. 

### 2.2. Toxicokinetic Characteristics Of Afg1

**Figure 1:**
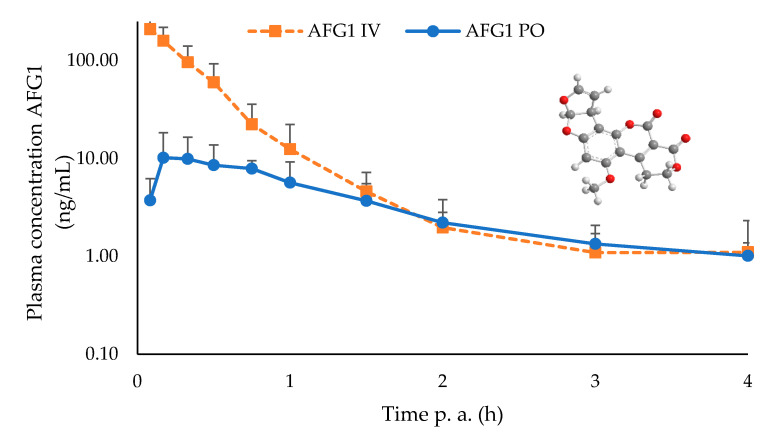
3D molecular structure (insert) and mean plasma concentration–time profile of AFG1 after oral (intra-crop bolus (PO)) and intravenous (IV) administration of 2 mg/kg bodyweight to broiler chickens (*n* = 10). Values are presented as the mean + or − SD. Plasma concentrations of AFG1 were quantified using UHPLC-MS/MS.

No clinical symptoms of intoxication were observed following the administration of 2 mg/kg BW of AFG1 PO or IV to the broilers. Plasma AFG1 concentration–time profiles, obtained after UHPLC-MS/MS analysis for oral and IV administrations in broiler chickens, are shown in Figure 1. 

Results of the mean toxicokinetic parameters of AFG1 are summarized in Table 2. Following oral administration, plasma AFG1 concentration increased rapidly in broiler chicken plasma to attain a maximum at 0.50 ± 0.30 h. Furthermore, AFG1 was rapidly eliminated after PO (T_1/2el_ = 1.36 ± 0.55 h) as well as after IV (T_l/2el_ = 0.50 ± 0.20 h) administration. The mean Vd and Cl values were 22.52 ± 20.32 L/kg and 28.50 ± 18.22 L/h/kg, respectively, after IV administration. Mean area under the curve (AUC)_0-t_ values were 12.83 ± 4.19 and 89.06 ± 36.94 h*ng/mL following PO and IV administration, respectively. Therefore, the oral bioavailability of AFG1 was 14.40 ± 4.70%.

### 2.3. UHPLC-HRMS Analysis

The plasma extracts were also injected onto a UHPLC-HRMS instrument to investigate the presence of eventual phase-I and phase-II metabolites for which no analytical standards are available. The same analytical column and mobile phases were used, as for the UHPLC-MS/MS analysis, but a slower gradient was applied (see Table 4). As can be seen in Figure S2B, this resulted in an increase in the retention time of AFG1 from ~4.4 min (UHPLC-MS/MS) to ~9.4 min (UHPLC-HRMS).

No peak at the elution zone of AFG1 was observed in the extracted ion chromatogram (XIC, 0.05 Da) at *m*/*z* = 329.0661 of a plasma sample that was taken prior to IV administration of AFG1 to a chicken (Figure S2A). The identity of the chromatographic peak at 9.4 min in a plasma sample of the same chicken, which was taken at 10 min after IV administration of AFG1 (dose: 2 mg/kg BW), could be confirmed based on the elemental composition of the ion with *m*/*z* = 329 in the low energy mass spectrum (proposed elemental composition: C_17_H_12_O_7_, observed accurate mass: *m*/*z* = 329.0676, mass error: 1.5 mDa or 4.5 ppm; Figure S2C) and the observed major fragment ions with *m*/*z* = 243.0646 and 311.0546 in the high-energy mass spectrum (Figure S2D). 

An untargeted approach was used to investigate the presence of metabolites, which means that certain transformations were added to the pathway profiling processing method, such as oxidation (+O), reduction (+H_2_), desaturation (−H_2_), hydration (+H_2_O), sulfation (+SO_3_), oxidation + desaturation (+O −H_2_), dehydration (−H_2_O), dihydrodiol formation (+H_2_O_2_), glucuronidation (+C_6_H_8_O_6_), and glutathione conjugation (+C_10_H_15_N_3_O_5_S). In Figure S3, XICs of the analysis of plasma samples that were taken at 10 min after IV administration (panel A) and at 30 min after the PO administration of AFG1 (panel B), are shown. As can be seen, AFG1 could be detected and confirmed in both plasma samples (see Table S3). No additional peaks were observed in the plasma sample after PO administration, indicating the absence of metabolites. In the plasma sample that was taken after IV administration, AFG1-related peaks were observed in the XIC at *m*/*z* = 311.0556 and 331.0818, which could be attributed to in-source fragmentation of AFG1 and to the ^14^C-isotope of the [M-H] + ion of AFG1. The identification of the peaks in the other XICs could not be confirmed, indicating the no AFG1-metabolites were present in this plasma sample. The same results were observed for all other plasma samples of the same chickens.

Quantification of the AFG1 concentration with the UHPLC-HRMS instrument was performed in plasma samples of 2 chickens after IV and PO administration using a targeted approach, i.e., the theoretical exact mass that was calculated on the basis of the chemical formula (*m*/*z* = 329.0661) was added to the processing method. As can be seen in Figure S4, a good correlation (r^2^ ≥ 0.9597) was found between the results obtained using both analytical techniques. 

### 2.4. Efficacy of the Calcinated Clay

**Figure 2:**
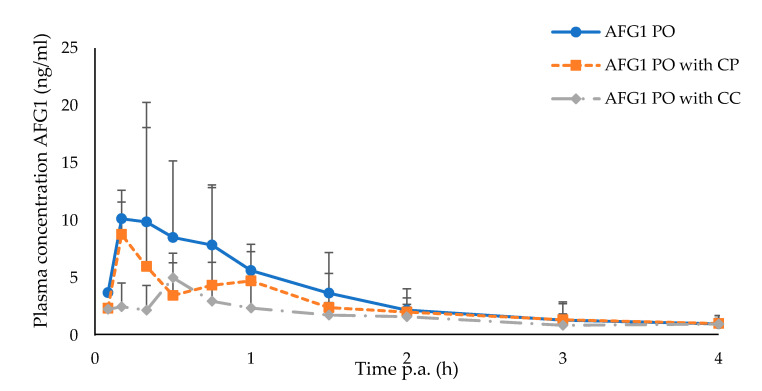
Mean plasma concentration–time profile of AFG1 in broiler chickens after oral (PO) administration (2 mg/kg BW), either with purified clay (CP, *n* = 10, orange curve) or with calcined clay (CC, *n* = 10, grey curve). Each profile represents the mean of 10 animals. Values are presented as the mean + or − SD. Plasma concentrations of AFG1 were quantified using UHPLC-MS/MS.

Figure 2 illustrates the plasma concentration–time profile of AFG1 after oral administration of AFG1 alone or in combination with CP and CC. The mean AUC_0-t_ was significantly lower after PO + CC administration compared to PO administration (*p* = 0.026) (Table 2). There was an average decrease of AUC_0-∞_ of approximately −47 % after CC administration compared to PO administration. Consequently, administering a calcined montmorillonite clay to broiler chickens resulted in a significantly decreased F of AFG1 (7.61 ± 4.76%) compared to birds that did not receive any mycotoxin detoxifier (PO, 14.40 ± 4.70%) (*p* = 0.026).

## 3. Discussion

In this study, an in vivo toxicokinetic study was performed in order to extend the knowledge of the absorption and oral bioavailability of AFG1 in broiler chickens and to evaluate the effect of calcination of montmorillonite clay on different toxicokinetic parameters of AFG1. The findings indicated that AFG1 is rapidly absorbed after oral administration (T_max_ = 0.50 ± 0.30 h). Similar research using murine models indicate that the absorption of AFs is a very fast process that follows first-order kinetic [46,52]. Corcuera et al. [47] found that the absorption of AFB1 in rats is very fast and that the molecule is rapidly metabolized in the liver. After exposure of rats to a single intratracheal or oral [H^3^]AFB1 dose, a peak of AFB1 plasma concentration was measured after one and three hours [53]. Gallo et al. [54] showed that AFs are quickly absorbed through the gastrointestinal tract of cows. Although rapid, AFG1 in the present study had a low oral bioavailability of 14.40 ± 4.70%. Besides the rapid but limited absorption, AFG1 was rapidly eliminated after PO (T_1/2el_ = 1.36 ± 0.55 h) as well as after IV (T_l/2el_ = 0.50 ± 0.20 h) administration. Both clearance and volume of distribution were high with a mean (±SD) Cl and Vd AFG1 of 28.50 ± 18.22 (L/h/kg) and 22.52 ± 20.32 (L/kg) in chickens, respectively. These results are in discordance with other studies that found that the elimination of AFs from the body is slower as compared to other mycotoxins [55]. In the case of laying hens, only 28% of the hens orally administered ^14^C-labelled AFB1 could be recovered during the first 24 h, and 71% were recovered within 7 days post-administration [49]. After IV administration of ^14^C-labelled AFB1 in mice, rats, and monkeys, the excretion of AFB1 was high during the initial 24 h [52]. The high Vd value inferred a relatively high tissue concentration of AFG1. In this context, Hussain et al. [50] showed that after 2 to 3 days of exposure of broiler chickens to a diet containing 0, 1.6, 3.2, and 6.4 mg AFB1/kg, AFB1 could be detected in livers of birds exposed to 1.6 mg/kg and higher for more than one week.

The developed UHPLC-MS/MS method used an Oasis^®^ Ostro 96-well plate (Waters, Zellik, Belgium) for sample clean-up, allowing protein precipitation and phospholipid removal in one action. Hence, it was possible to extract 96 plasma samples simultaneously. By combining this simple and straightforward sample preparation procedure with a gradient elution of 10 min on the UHPLC-MS/MS instrument, a large number of samples (*n* ≥ 96) could be analyzed within a 24 h period, which was an advantage if a large number of samples had to be analyzed as a part of toxicokinetic studies. The method was successfully validated and allowed the quantification of AFG1 in broiler plasma samples over a range of 0.5–200 ng/mL. The LOQ value of 0.5 ng/mL was low enough to allow accurate quantification of AFG1 in plasma samples that were taken from broiler chickens after IV or PO administration (dose: 2 mg/kg BW). 

The knowledge of metabolites of AFG1 in broiler chicken is limited; therefore, the plasma samples obtained during the toxicokinetic study after oral and intravenous administration were analyzed using UHPLC-HRMS to identify the presence of phase I and II metabolites. The UHPLC-MS/MS method was transferred to a UHPLC-HRMS instrument with some modifications in the chromatographic gradient to increase the retention time of AFG1 and allow a better separation of potential metabolites. Using a non-targeted pathway profiling approach, no relevant metabolites were found by UHPLC-HRMS. This can be attributed to the short exposure to AFG1. In contrast, in a recent paper, Slobodchikova et al. [56] characterized phase I and phase II glucuronide metabolites of in vitro microsomal incubation. These authors showed the presence of one hydroxy metabolite of AFG1, which can be identified as AFGM1 metabolite. In addition, the good correlation between quantitative results obtained with both the UHPLC-MS/MS and UHPLC-HR-MS instruments demonstrated the potential of the latter technique for use in the field of quantitative analysis in the future. 

In the current study, AFG1 was also administered with purified and calcined montmorillonite to test the effectiveness of calcination of the clay mineral in adsorbing AFG1. A significant decrease of 47% in AUC was observed after the CC administration compared to PO administration indicating that the calcined clay was effective in binding AFG1. Lauwers et al. [57] stated that the detoxifier was effective in binding mycotoxins when the AUC was significantly lower when compared to the AUC without detoxifier in plasma and urine, whereas the k_el_ was not significantly different between the three groups. In addition, no significant difference was observed in maximum concentration and time to maximum concentration. A recent study showed a significant influence of a mycotoxin-detoxifying agent on the AUC of AFB1 in broiler chickens [57]. In the same study, the authors did not observe any significant difference in C_max_ and T_max_. In our study, the F value was significantly decreased by 47% after the administration of calcined clay. This could be attributed to the adsorption of AFG1 by the calcined clay in the gastrointestinal tract. Previously, it has been demonstrated by in vitro study that calcination improved the adsorption of AFs and mainly of AFG1 and AFG2 at pH 3 and pH 7 of the gastrointestinal tract [38]. The in vitro binding affinity is consistent with the in vivo results observed here and supports the hypothesis that calcination improves the aflatoxin-binding capacity of montmorillonite clay, which is a key property involved in the toxicity-alleviating effects. 

## 4. Conclusions

In this research, the toxicokinetic characteristic and bioavailability of AFG1 in broiler chicken were interpreted for the first time. AFG1 is rapidly absorbed after oral administration and rapidly eliminated after oral as well as IV administration. The oral bioavailability was low (18.43%), which may be due to the rapid transport of the toxin through the alimentary tract of the chickens. In addition, the in vivo efficacy of calcined montmorillonite in binding AFG1 was demonstrated for the first time. Calcination reduced the bioavailability, thus avoiding its toxic effects. Notwithstanding the high adsorption of AFs by calcinated clay, little is known about the unspecific binding, which is a major consideration. Mycotoxin binders may indeed also adsorb nutrients, micronutrients, and/or veterinary drugs [58,59]. Therefore, in vivo safety testing of the studied binder is needed. Moreover, research evaluating the effect of calcination on other types of clay on other mycotoxins should be done.

## 5. Materials and Methods 

### 5.1. Standard, Reagents, and Solutions

The standard of AFG1, used for both plasma analysis and the animal experiment, was obtained from Fermentek Ltd. For the LC-MS/MS analysis, the internal standard (IS) ^13^C_17_-AFG1 was obtained as a 0.5 µg/mL solution in acetonitrile (ACN) from Biopure (Tulln, Austria). All standards were kept at ≤−15 °C. Methanol (MeOH) and ACN were of ultra-liquid chromatography (ULC-MS) grade and were purchased from Biosolve (Valkenswaard, The Netherlands). UPLC-grade water was obtained from a Milli-Q Reference A+ system (Merck, Overijse, Belgium). Formic acid (FA) of analytical grade was purchased from VWR (Leuven, Belgium). Dimethyl sulfoxide (DMSO) used for the animal experiment was of analytical grade and was obtained from Filterservice (Eupen, Belgium). Oasis^®^ Ostro protein precipitation and phospholipid removal 96-well plates (25 mg), 96-well collector plates, and polypropylene mat caps for 96-well plates were purchased from Waters (Zellik, Belgium). A Tunisian clay was collected from Jebel Aïdoudi (El Hamma, Gabes, Tunisia) and was purified to obtain the purified clay (CP) as described [38]. The thermally treated clay (CC) was obtained by calcination of the purified clay at 550 °C for 5 h. The chemical composition of both clays did not change after calcination and was characterized by a high calcium content and a low percentage of sodium oxide. The analysis of the studied clay with X-ray diffraction exhibited that the studied clay was composed mostly of calcic smectite [38].

### 5.2. Preparation of Standard Solutions

For the animal trial, a stock solution (SS) of AFG1 (8 mg/mL) was prepared in DMSO. For the plasma analysis, a SS of AFG1 (0.2 mg/mL) was prepared in ACN. Working solutions of AFG1 (WS) at concentrations of 1000 ng/mL, 100 ng/mL, 10 ng/mL, 1 ng/mL, and 0.1 ng/mL were prepared by appropriate dilution of the SS in ACN. For the IS, a working solution (WS_IS_) containing ^13^C_17_-AFG1 at a concentration of 10 ng/mL was prepared in ACN. The SS and all the WS were stored at ≤−15 °C. 

### 5.3. Animals and Toxicokinetic Study Design

A total of forty, 3 weeks-old healthy broiler chickens (Ross 308, ♂/♀, 20/20) were obtained from a commercial breeder (Moerbeke, Belgium). A lighting cycle of 18 h of light and 6 h of darkness was applied. The temperature regime was adjusted to the changing needs of the animals according to their age. During the one-week acclimatization period, all animals were group-housed in a floor pen of 4 m^2^. Mycotoxin control feed and water were given *ad libitum* during the acclimatization and experimental period. 

Commercially available broiler mash feed was obtained from AVEVE (Merksem, Belgium). This feed was analyzed for the presence of the following mycotoxins: 3 and 15-acetyldeoxynivalenol (3- and 15-ADON), deoxynivalenol (DON), T2 and HT2-toxin, AFB1, AFB2, AFG1, and AFG2, fumonisin B1 + B2 (FB1 and FB2), zearalenone (ZEN), nivalenol (NIV), cytochalasin E, and ochratoxin A (OTA), by a multi-mycotoxin liquid chromatography-tandem mass spectrometry (LC-MS/MS) method (Primoris, Zwijnaarde, Belgium). This control feed contained 125 µg/kg of DON and 44.4 µg/kg of FB1 + FB2; the levels were below the maximum guidance level allowed by the EU regulations [60].

After one-week acclimatization, animals were weighed and subsequently randomly distributed based on sex and body weight into four groups of 10 animals (♂/♀, 5/5). During the experimental phase of the study, animals were housed per group in a floor pen of 2 m². Eight hours before the mycotoxin bolus administration, the animals were deprived of feed, until 3 h post-administration. 

Animals of the first group were administered a bolus AFG1 (2 mg/kg BW) by IV injection in the wing vein. The second group was administered AFG1 (2 mg/kg BW) orally by an intra-crop bolus (PO) followed by 4 mL of sterile distilled water (PO control group). The third group was administered first a bolus of AFG1 PO (2 mg/kg BW) then an intra-crop bolus of purified clay (CP, 1 g/kg BW, suspended in 2 mL of sterile distilled water), followed by 2 mL of sterile distilled water to flush the crop tube. The fourth group received first a bolus of AFG1 PO (2 mg/kg BW) then an intra-crop bolus of calcinated clay (CC, 1 g/kg BW, suspended in 2 mL of sterile distilled water), followed by 2 mL of sterile distilled water to flush the crop tube. Before the administration of the mycotoxin, the PO and IV AFG1 bolus solution were prepared instantaneously by further dilution of the AFG1 SS with water (PO) or physiological saline (IV). After administration of AFG1 with or without clay, 0.5 mL of blood was collected via the leg vein at 0 (just before administration), 0.08, 0.17, 0.33, 0.5, 0.75, 1, 1.5, 2, 3, 4, 6, 8, 12, and 24 h post-administration in heparinized tubes (Vacutest Kima, Novolab, Geraardsbergen, Belgium). The samples were centrifuged (3724 × g, 10 min, 4 °C), and aliquots of plasma (100 µL) were stored at ≤−15 °C until analysis. 

The animal experiment was approved by the ethical committee of the Faculty of Veterinary Medicine and the Faculty of Bioscience Engineering of Ghent University (EC 2019/70, approval date: 17 October 2019). 

### 5.4. Plasma AFG1 Analysis

#### 5.4.1. Sample Pre-Treatment

To 100 µL of chicken plasma, 25 µL of IS working solution (10 ng/mL) and 100 µL of ACN were added, followed by a vortex mixing step and further equilibration at room temperature for 5 min. The samples were loaded onto an Oasis^®^ Ostro 96-well plate (25 mg), followed by the addition of 300 µL of 1% FA in ACN. The sample was mixed by aspiration using a pipette (5 times) and passed through the 96-well plate while the vacuum was applied (15 mm Hg) for 5 min. The filtrate was collected in a 96-well collector plate and evaporated under a gentle nitrogen stream (~40 °C). The dry residue was reconstituted in 200 µL of water/methanol (MeOH) (50/50, *v*/*v*), followed by vortex mixing for 15 s. After covering the 96-well collector plate with a polypropylene mat cap, an aliquot 5.0 µL was injected onto the UHPLC-MS/MS instrument. For samples with a concentration out of the calibration curve, the injection volume was reduced to 0.5 or 1.0 µL. 

#### 5.4.2. UHPLC-MS/MS Analysis 

The UHPLC-MS/MS instrument consisted of an Acquity UPLC^®^ H-Class Quaternary Solvent Manager and Flow-Through-Needle Sample Manager with temperature-controlled tray and column oven (Waters, Zellik, Belgium). The column was an Acquity UPLC^®^ HSS T3 (Waters, Zellik, Belgium), 100 mm × 2.1 mm i.d., dp: 1.8 µm, associated with an Acquity HSS T3 1.8 μm Vanguard pre-column (Waters, Zellik, Belgium). Chromatography was performed using water and methanol as mobile phase A and B, respectively, in gradient elution as displayed in Table 3. The flow-rate of the mobile phase was set at 0.3 mL/min. The temperatures of the column oven and autosampler tray were set at 40 °C and 8 °C, respectively.

The UHPLC column effluent was sent to a Xevo TQ-S^®^ MS/MS system, equipped with an electrospray ionization (ESI) probe that was operated in the positive mode (all from Waters, Zellik, Belgium). The UHPLC eluent was directed to the mass spectrometer from 3.0 to 6.0 min, using a divert valve. MS/MS instrument parameters were determined by direct infusion of WS of 100 ng/mL of AFG1 and the IS, respectively, at a flow rate of 10 µL/min and in combination with the mobile phase A/B (50/50, *v*/*v*) at a flow-rate of 200 µL/min. The following parameters were used: capillary voltage: 3.2 kV, source offset: 50 V, source temperature: 150 °C, desolvation temperature: 600 °C, desolvation gas: 800 L/h, cone gas: 150 L/h, nebuliser pressure: 6.9 bar, low mass resolution 1 and 2: 2.8, high mass resolution 1 and 2: 15.00, respectively, ion energy 1 and 2: 0.2 and 0.8, respectively, collision gas flow: 0.15 mL/min.

MS/MS acquisition was performed in the multiple reaction monitoring (MRM) mode and an overview of the MRM transitions for AFG1 and the IS is given in supplementary Table S1. Data acquisition and processing were performed using the MassLynx v.4.1 software (Waters, Zellik, Belgium).

#### 5.4.3. In-House Method Validation

The developed UHPLC-MS/MS method was validated in-house for AFG1 based on a protocol that was previously described by De Baere et al. [61]. Spiked blank plasma samples that were obtained from healthy, untreated chickens were used for method validation. The following parameters were evaluated: linearity, accuracy, precision, the limit of quantification (LOQ), the limit of detection (LOD), and carry-over, according to the recommendations and guidelines defined by the European Community and with criteria described in the literature [51,61,62,63,64,65].

Linearity: Linearity was evaluated over a concentration range between 0.05 and 200 ng/mL using matrix-matched calibration curves. The correlation coefficients (r) and goodness-of-fit coefficients (g) were determined and had to comply with the following limits: r ≥ 0.99 and g ≤ 20%, respectively. 

Accuracy and precision: Accuracy and precision were determined by analyzing three sets of 6 spiked blank samples in the same run at the following concentration levels of AFG1: 0.5 ng/mL, 5.0 ng/mL, and 50.0 ng/mL. The between-run accuracy and precision were evaluated in a similar way by analyzing at least 3 blank samples spiked at the same concentration levels on three different days. The acceptance criteria for accuracy were met at all concentration levels (Supplementary Table S3). The precision was evaluated by the determination of the relative standard deviation (RSD), which had to be below the RSD_max_ value. 

Limit of quantification: The LOQ was established as the lowest point of the calibration curve and was set at 0.05 ng/mL.

Limit of detection: The LOD was calculated as 3 times the standard deviation of the y-intercept divided by the average slope of three independent calibration curves. 

Carry-over: The absence/presence of carry-over on the UHPLC-MS/MS instrument was evaluated by analyzing the reconstitution solvent injected after the highest calibrator sample.

Specificity: to investigate the specificity of the analytical method, a blank plasma sample was extracted and analyzed in each analytical batch. 

### 5.5. Plasma Phase I and Phase II Metabolites of AFG1

UHPLC-HRMS analysis was performed on plasma samples from four animals after PO administration and five animals after IV administration.

The UHPLC-HRMS instrument consisted of an Acquity I-Class UPLC coupled to a Synapt G2-S*i*HDMS instrument (Waters, Zellik, Belgium) and was used to identify potential phase-I and phase-II metabolites of AFG1 in incurred chicken plasma samples. The same analytical column and mobile phases were used as described in Table 4. The flow rate was set at 0.3 mL/min. The temperatures of the column oven and autosampler tray were 40 °C and 8 °C, respectively. A standard solution of AFG1 was infused by a syringe to optimize HR-MS instrument parameters. The following HR-MS parameters were finally selected: capillary voltage, 2.70 kV; sampling cone voltage, 30.0 V; source offset, 80.00 V; source temperature, 150 °C; desolvation temperature, 550 °C; cone gas flow, 50 L/h; desolvation gas flow, 800 L/h; nebulizer gas flow, 6.50 bar; lock spray capillary voltage, 2.0 kV. Data acquisition was performed between 0.5 and 11.5 min in the positive ESI ionization mode using MS^E^ continuum scanning. The following time-of-flight MS settings were used: low mass, 50 Da; high mass, 950 Da; scan time, 0.15 s; data format, continuum; collision energy (CE): low energy trap and transfer CE: off, high energy trap CE: ramp between 10 to 60 V, transfer CE: off. Leucine encephalin (200 pg/µL) was used as a lock mass component. The lock spray was acquired, but no correction was applied during HR-MS acquisition. The lock spray settings were: scan time, 0.15 s; interval, 30 s; scans to average, 3; mass window, 0.5 Da. Data processing and lock mass correction (*m*/*z* 556.2765) was performed using the Unify 1.8 software (Waters, Zellik, Belgium). The identification of AFG1 was based on retention time (target T_R_ tolerance: 0.1 min) and mass (target mass tolerance: 10 ppm). The examination of phase I and phase II metabolites of AFG1 was performed using a pathway profiling approach. The following transformations were looked for: oxidation (+O), reduction (+H_2_), desaturation (−H_2_), hydration (+H_2_O), sulfation (+SO_3_), oxidation +desaturation (+O −H_2_), dehydration (−H_2_O), dihydrodiol formation (+H_2_O_2_), glucuronidation (+C_6_H_8_O_6_), glutathione conjugation (+C_10_H_15_N_3_O_5_S).

### 5.6. Toxicokinetic and Statistical Analysis

Non-compartmental toxicokinetic modeling was carried out using Phoenix (Princeton, NJ, USA). Plasma concentrations under the LOQ were not considered. The most important toxicokinetic parameters were calculated for IV and PO administration: area under the plasma concentration–time curve from time 0 to 8 h (AUC_0-t_), area under the plasma concentration–time curve from 0 to infinity (AUC_0-∞_), maximal plasma concentration (C_max_), plasma concentration at time 0 (C_0_), time to maximal plasma concentration (T_max_), elimination rate constant (k_el_), elimination half-life (T_1/2el_), clearance (Cl) and volume of distribution (Vd). The absolute oral bioavailability (F), expressed as a percentage, was calculated according to the formula:

F = (AUC_0-∞_ PO / AUC_0-∞_ IV) × 100 (1)

All toxicokinetic parameters were compared with a Tukey HSD test (SPSS 26, IBM, New York, NY, USA). The level of significance was set at 0.05. Graphs were obtained with Microsoft Office (office 365). The 3D molecular structure of AFG1 was obtained with Chem3D v15 (shareware).

## Figures and Tables

**Table 1:** Results of the evaluation of linearity (goodness-of-fit coefficient (g), correlation coefficient (r)), limit of quantitation (LOQ), and limit of detection (LOD) for the analysis of aflatoxin G1 (AFG1) in broiler chickens’ plasma.

Matrix	Calibration Range (ng/mL)	*g* (%) ^a^	*r* ^a^	LOQ (ng/mL)	LOD (ng/mL)
Broiler Chicken plasma	0.50–200	4.50 ± 1.48	0.9987 ± 0.0008	0.50	0.16

^a^ Acceptance criteria: *g* ≤ 20%, *r* ≥ 0.99.

**Table 2:** Mean toxicokinetic parameters of AFG1 determined after intravenous (IV) and oral (PO) administration (2 mg/kg BW) to broiler chickens, either with purified clay (CP, *n* = 10) or with calcined clay (CC, *n* = 10).

Toxicokinetic Parameters		Route of Administration			
	IV	PO			
		PO	PO + CP	PO + CC	*p*-Value
AUC_0-t_ (h*ng/mL)	89.06 ± 36.94	12.83 ± 4.19 ^a^	11.36 ± 5.10 ^a,b^	6.78 ± 4.24 ^b^	0.026
AUC_0-∞_ (h*ng/mL)	89.86 ± 36.73	15.10 ± 4.82 ^a^	14.73 ± 5.08 ^a,b^	9.06 ± 5.03 ^b^	0.041
C_max_ (ng/mL)	_	11.01 ± 5.32	10.41 ± 7.50	13.59 ± 14.95	0.760
C_0_ (ng/mL)	274.62 ± 144.49	_	_	_	_
T_max_ (h)	_	0.50 ± 0.30	0.48 ± 0.36	0.48 ± 0.34	0.987
k_el_ (h-1)	1.53 ± 0.42	0.57 ± 0.17	0.35 ± 0.26	0.55 ± 0.36	0.158
T_1/2el_ (h)	0.50 ± 0.20	1.36 ± 0.55	2.15 ± 1.61	2.49 ± 0.30	0.498
Cl (L/h/kg)	28.50 ± 18.22	_	_	_	_
Vd (L/kg)	22.52 ± 20.32	_	_	_	_
F (%)	100	14.40 ± 4.70 ^a^	12.75 ± 5.73 ^a,b^	7.61 ± 4.76 ^b^	0.026

AUC_0−t_, area under the plasma concentration−time curve from time 0 to last concentration > LOQ; AUC_0−∞_, area under the plasma concentration−time curve from time 0 to infinity; C_max_, maximal plasma concentration; C_0_, plasma concentration at time 0; t_max_, time to maximal plasma concentration; k_el_, elimination rate constant; t_1/2el_, elimination half-life; Cl, total body clearance; Vd, volume of distribution; F, oral bioavailability. Values in each row indicated by different letters ^(a,b)^ are significantly different (*p* ≤ 0.05). Values are presented as the mean ± SD.

**Table 3:** Gradient elution for the UHPLC-MS/MS separation.

Time (min)	% Mobile Phase A	% Mobile Phase B
0–1.0	80	20
3.0	-	90
3.0–7.0	10	90
7.3	-	90
7.3–10.0	80	20

**Table 4:** Gradient elution for the UHPLC-HRMS separation.

Time (min)	% Mobile Phase A	% Mobile Phase B
0–2.0	90	10
4.0	-	30
4.0–6.0	70	30
8	-	50
8.0–10.0	50	50
12.0	-	70
12.0–14.0	30	70
14.2	-	90
14.2–16.5	10	90
17.0	-	10
17.0–20.0	90	10

## Supplementary Materials

The following are available online at https://www.mdpi.com/2072-6651/12/10/660/s1, Table S1: MRM transitions and MS/MS parameters for AFG1 and the internal standard, ^13^C_17_-AFG1; Table S2: Results of the within-run and between-run precision and accuracy evaluation for the analysis of aflatoxin G1 in chicken plasma; Table S3: Results of the investigation of the UHPLC-HRMS extracted ion chromatograms (XIC) of a plasma sample taken at (A) 10 min after intravenous administration and (B) 30 min after oral administration of 2 mg AFG1/kg BW for possible phase I and phase II metabolites; Figure S1: UHPLC-MS/MS chromatogram of (A) a blank plasma sample, (B) a blank plasma sample spiked at the LOQ level (AFG1 concentration: 0.50 ng/mL), a plasma sample taken at (C) 10 min after intravenous administration (AFG1 concentration: 101.7 ng/mL) and (D) 30 min after oral administration of 2 mg AFG1/kg BW (AFG1 concentration: 12.0 ng/mL); Figure S2: UHPLC-HRMS extracted ion chromatogram (XIC) at *m*/*z* = 329.0661 of a plasma sample taken (A) before and (B) at 10 min after intravenous administration of 2 mg/kg BW, showing a peak of AFG1 at Tr = 9.42 min (concentration: 89.2 ng/mL); (C) low-energy spectrum of the peak at Tr = 9.42 min, showing the [M-H]+ ion of AFG1 (observed accurate mass at *m*/*z* = 329.0699, mass error: 1.5 mDa or 4.5 ppm); (D) high-energy spectrum of the same peak, showing the two major fragment ions of AFG1 at *m*/*z* = 243.0646 and 311.0546; Figure S3: UHPLC-HRMS extracted ion chromatograms (XIC) of a plasma sample taken at (A) 10 min after intravenous administration (AFG1 concentration: 89.2 ng/mL) and (B) 30 min after oral administration of 2 mg AFG1/kg BW (AFG1 concentration: 11.7 ng/mL). The following mass-to-charge (*m*/*z*) values, corresponding with the theoretical exact mass of the protonated molecular ions [M-H]+, were extracted from the total ion chromatogram: (a) parent AFG1, C_17_H_12_O_7_: 329.0661; (b) oxidation (+O), C_17_H_12_O_8_: 345.0610; (c) reduction (+H_2_), C_17_H_14_O_7_: 331.0818: (d) desaturation (−H_2_), C_17_H_10_O_7_: 327.0505; (e) hydration (+H_2_O), C_17_H_14_O_8_: 347.0767; (f) sulfation (+SO_3_), C_17_H_12_O_10_S: 409.0229; (g) oxidation + desaturation (+O −H_2_), C_17_H_10_O_8_: 343.0454; (h) dehydration (−H_2_O), C_17_H_10_O_6_: 311.0556; (i) dihydrodiol formation (+H_2_O_2_), C_17_H_14_O_9_: 363.0716; (j) glucuronidation (+C_6_H_8_O_6_), C_23_H_20_O_13_: 505.0982; (k) glutathione conjugation (+C_10_H_15_N_3_O_5_S), C_27_H_27_N_3_O_12_S; 618.1394; Figure S4: Correlation between the AFG1 plasma concentrations in 2 chickens that received an (A) oral and (B) intravenous administration of 2 mg/kg BW, after quantitative analysis using the UHPLC-MS/MS and UPLC-HRMS technique, respectively.
