# Supplementary material for: Calcination Improves the In Vivo Efficacy of a Montmorillonite Clay to Bind Aflatoxin G1 in Broiler Chickens: A Toxicokinetic Approach
Source: Toxins (Basel). 2020 Oct 18;12(10):660. doi: 10.3390/toxins12100660 (PMC7603272; doi:10.3390/toxins12100660)
Supplement: Supplementary file 1 [file toxins-12-00660-s001.pdf]

Supplementary Materials: Calcination Improves the in Vivo Efficacy of a Montmorillonite Clay to Bind Aflatoxin G1 in Broiler Chickens: A Toxicokinetic Approach

Roua Rejeb, Siegrid De Baere, Mathias Devreese, Richard Ducatelle, Siska Croubels, Madiha Hadj Ayed, Achraf Ghorbal and Gunther Antonissen

Table S1. MRM transitions and MS/MS parameters for AFG1 and the internal standard, $^{13}C_{17}$-AFG1.

Analyte	Precursor ion (m/z)[a]	Product ions (m/z)	CE[b] (eV)	Cone (V)	Retention time (min)
**AFG1**	329.1	243.0[c]	23	35	4.38
		311.1	18	35	
**$^{13}C_{17}$-AFG1**	346.1	257.1[c]	25	40	4.38
		328.1	20	40	

Note: [a] m/z = mass-to-charge ratio, [b] CE = collision energy, [c] ion used for quantification.

Table S2. Results of the within-run and between-run precision and accuracy evaluation for the analysis of aflatoxin G1 in chicken plasma.

Theoretical concentration (ng/mL)	Mean concentration ± SD (ng/mL)	Precision, RSD (%)	Accuracy (%)
0.50 [a]	0.50 ± 0.01	2.1	-0.6
0.50 [b]	0.53 ± 0.07	13.0	6.2
5.00 [a]	4.88 ± 0.14	3.0	-2.4
5.00 [b]	5.20 ± 0.68	13.0	4.1
50.0 [a]	48.2 ± 1.8	3.7	-3.6
50.0 [b]	48.3 ± 2.2	4.5	-3.4

Note: [a] Within-run accuracy and precision (n=6); [b] Between-run accuracy and precision (n= 3 x 6); SD: standard deviation; RSD: relative standard deviation; Acceptance criteria: accuracy: < 1 ng/mL: -50% to +20%, ≥ 1 to < 10 ng/mL: -40% to +20%, ≥ 10 to < 100 ng/mL: -30% to +10%, within-run precision (RSD_{max}): < 1 ng/mL: 30 %, ≥1 to < 10 ng/mL: 25.0%, ≥ 10 ng/mL: 15.0%, between-run precision: < 1 ng/mL: 45%, ≥ 1 to < 10 ng/mL: 32%, ≥ 10 to < 100 ng/mL: 23%, ≥100 ng/mL: 16% [VICH GL49].

Table S3. Results of the investigation of the UHPLC-HRMS extracted ion chromatograms (XIC) of a plasma sample taken at (A) 10 min after intravenous administration and (B) 30 min after oral administration of 2 mg AFG1/kg BW for possible phase I and phase II metabolites.

	Concentration (ng/mL)	Retention time (min)	Calculated monoisotopic mass (m/z)	Observed mass (m/z)	Mass error (mDa)	Mass error (ppm)	Molecular ion
Sample A : AFG1	89.2	9.40	329.0661	329.0676	1.5	4.5	[M-H]+
Sample B : AFG1	11.7	9.40	329.0661	329.0659	-0.2	-0.6	[M-H]+

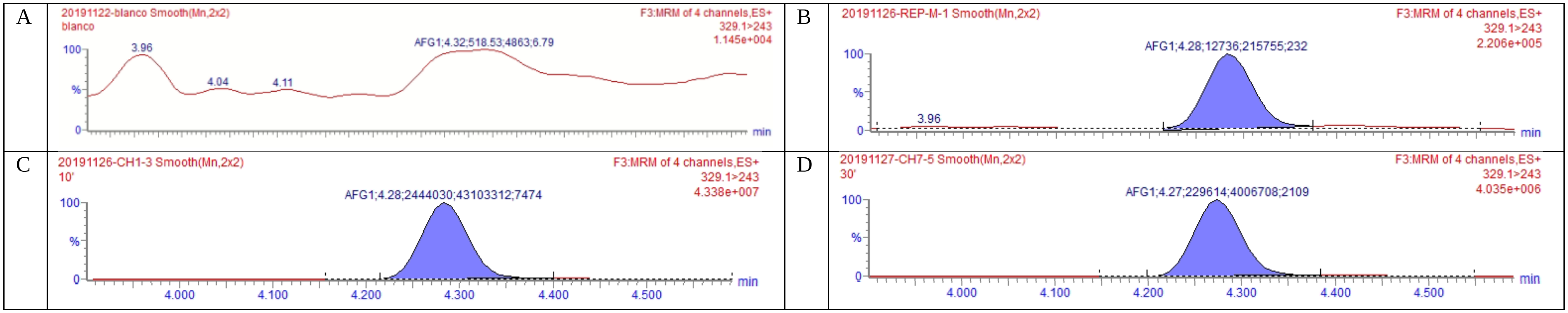

Figure S1. UHPLC-MS/MS chromatogram of (A) a blank plasma sample, (B) a blank plasma sample spiked at the LOQ level (AFG1 concentration: 0.50 ng/ml), a plasma sample taken at (C) 10 min after intravenous administration (AFG1 concentration : 101.7 ng/ml) and (D) 30 min after oral administration of 2 mg AFG1/kg BW (AFG1 concentration : 12.0 ng/ml).

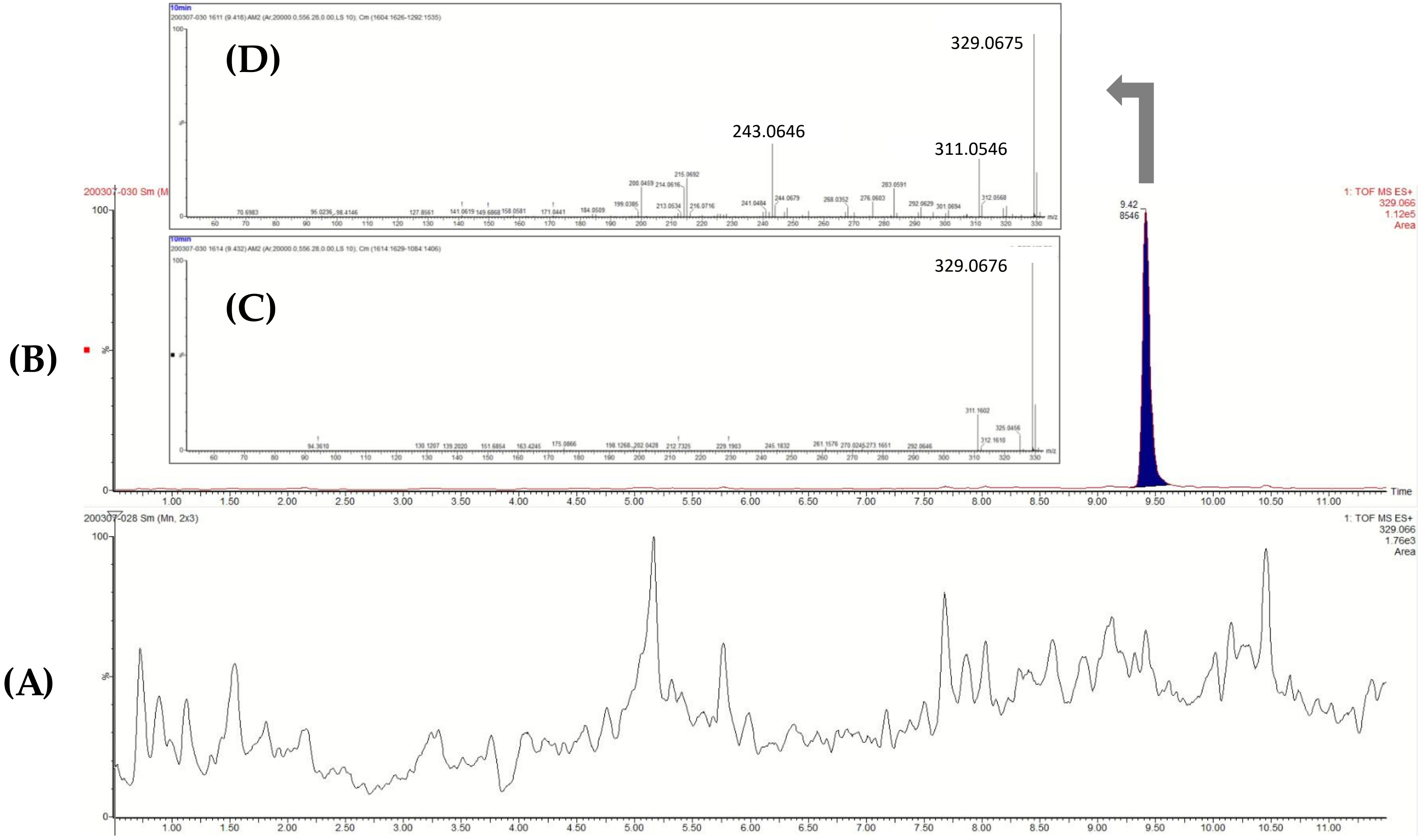

Figure S2. UHPLC-HRMS extracted ion chromatogram (XIC) at m/z = 329.0661 of a plasma sample taken (A) before and (B) at 10 min after intravenous administration of 2 mg/kg BW, showing a peak of AFG1 at Tr = 9.42 min (concentration : 89.2 ng/ml); (C) low energy spectrum of the peak at Tr = 9.42 min, showing the [M-H]+ ion of AFG1 (observed accurate mass at m/z = 329.0699, mass error: 1.5 mDa or 4.5 ppm); (D) high energy spectrum of the same peak, showing the two major fragment ions of AFG1 at m/z = 243.0646 and 311.0546.

(A)

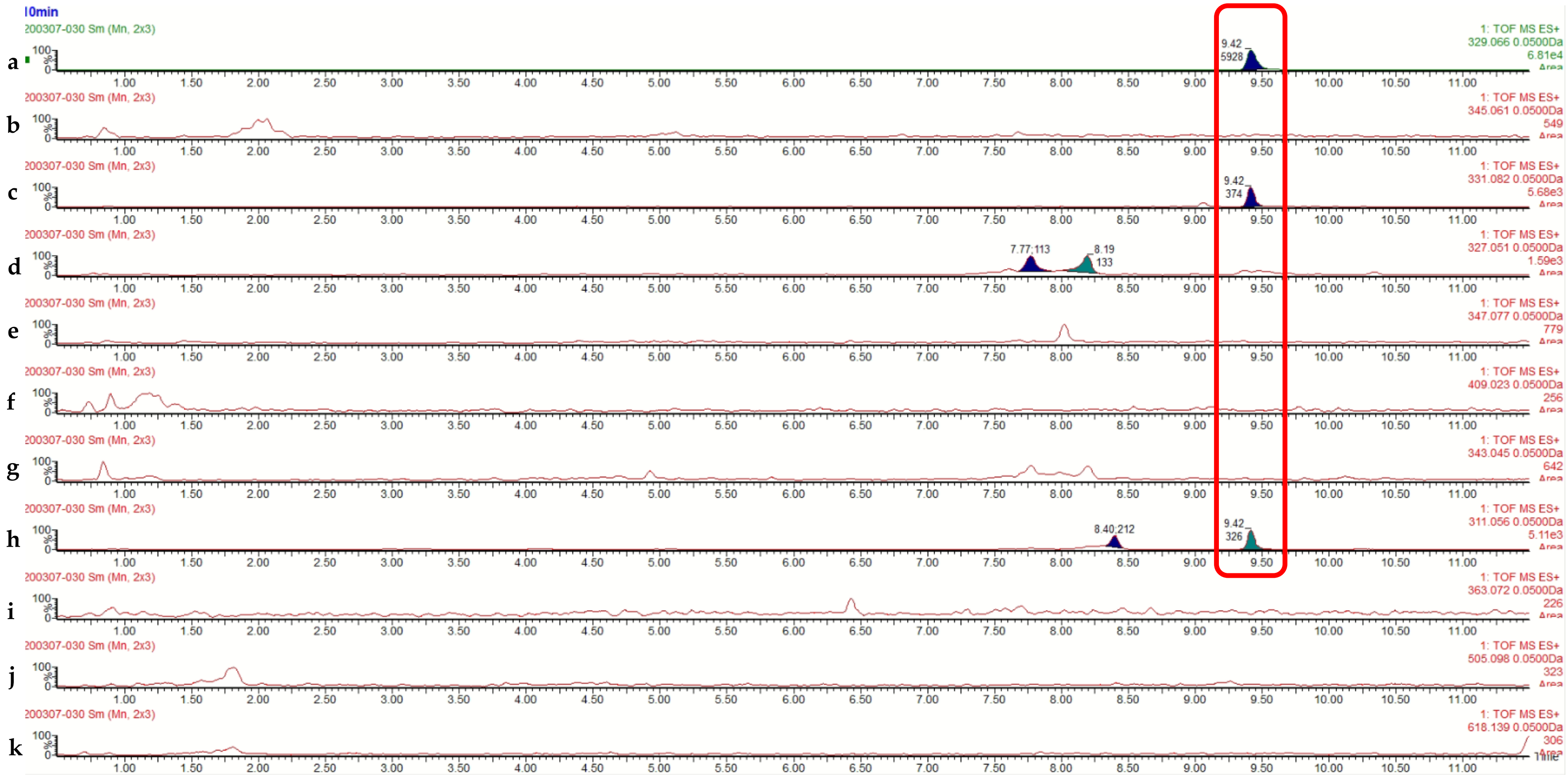
10min
200307-030 Sm (Mn, 2x3)
a
1: TOF MS ES+
329.066 0.0500Da
6.81e4
9.42
5928
b
1: TOF MS ES+
345.061 0.0500Da
549
c
1: TOF MS ES+
331.082 0.0500Da
5.68e3
9.42
374
d
1: TOF MS ES+
327.051 0.0500Da
1.59e3
7.77;113
8.19
133
e
1: TOF MS ES+
347.077 0.0500Da
779
f
1: TOF MS ES+
409.023 0.0500Da
256
g
1: TOF MS ES+
343.045 0.0500Da
642
h
1: TOF MS ES+
311.056 0.0500Da
5.11e3
8.40;212
9.42
326
i
1: TOF MS ES+
363.072 0.0500Da
226
j
1: TOF MS ES+
505.098 0.0500Da
323
k
1: TOF MS ES+
618.139 0.0500Da
306
Time

(B)

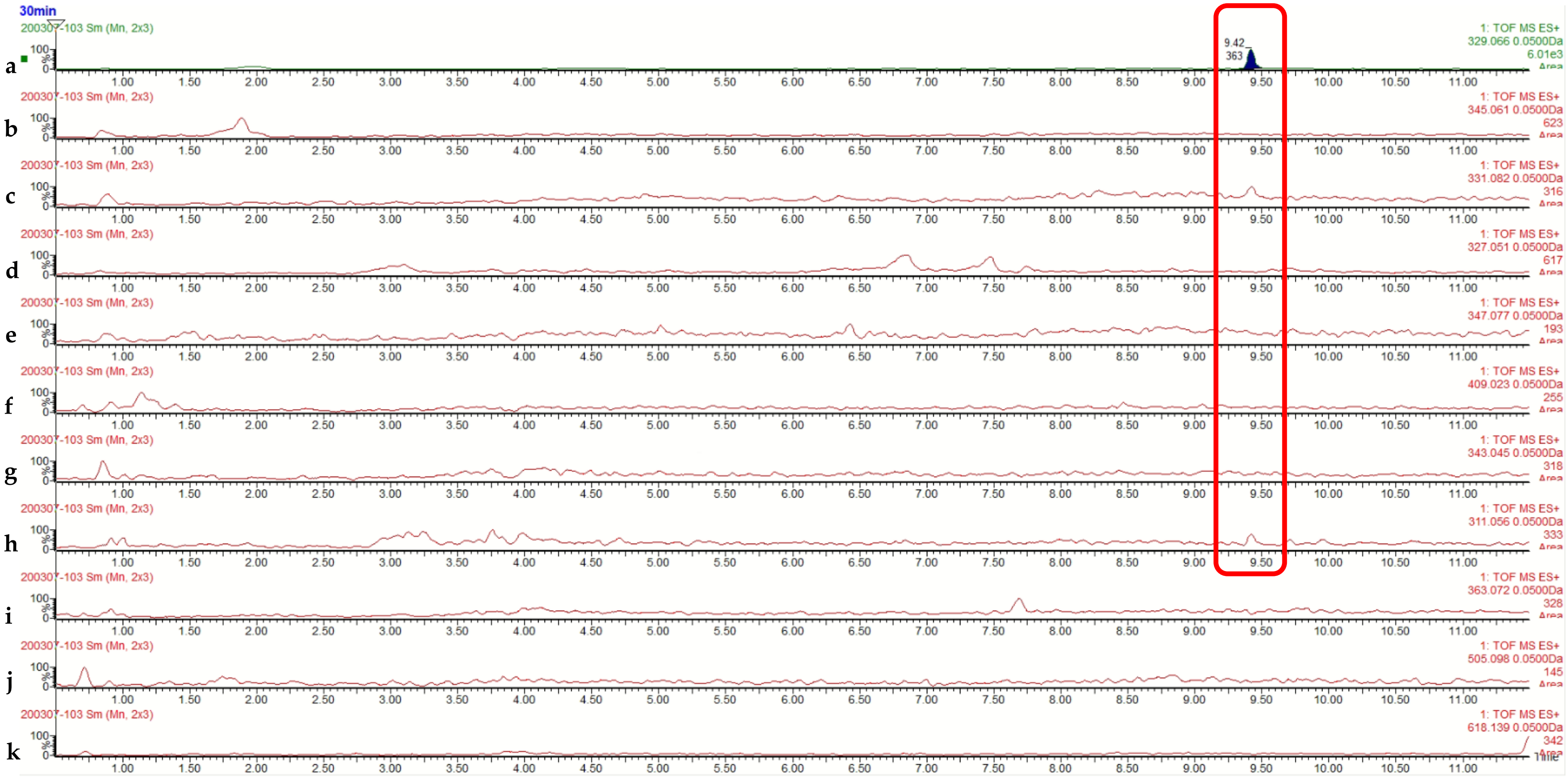

Figure S3. UHPLC-HRMS extracted ion chromatograms (XIC) of a plasma sample taken at (A) 10 min after intravenous administration (AFG1 concentration : 89.2 ng/ml) and (B) 30 min after oral administration of 2 mg AFG1/kg BW (AFG1 concentration : 11.7 ng/ml). The following mass-to-charge (m/z) values, corresponding with the theoretical exact mass of the protonated molecular ions [M-H]+, were extracted from the total ion chromatogram: (a) parent AFG1, $C_{17}H_{12}O_7$: 329.0661; (b) oxidation (+O), $C_{17}H_{12}O_8$: 345.0610; (c) reduction (+H_2), $C_{17}H_{14}O_7$: 331.0818: (d) desaturation (-H_2), $C_{17}H_{10}O_7$: 327.0505; (e) hydration (+H_2O), $C_{17}H_{14}O_8$: 347.0767; (f) sulfation (+SO_3), $C_{17}H_{12}O_{10}S$: 409.0229; (g) oxidation + desaturation (+O-H_2), $C_{17}H_{10}O_8$: 343.0454; (h) dehydration (-H_2O), $C_{17}H_{10}O_6$: 311.0556; (i) dihydrodiol formation (+H_2O_2), $C_{17}H_{14}O_9$: 363.0716; (j) glucuronidation (+$C_6H_8O_6$), $C_{23}H_{20}O_{13}$: 505.0982; (k) glutathione conjugation (+$C_{10}H_{15}N_3O_5S$), $C_{27}H_{27}N_3O_{12}S$; 618.1394.

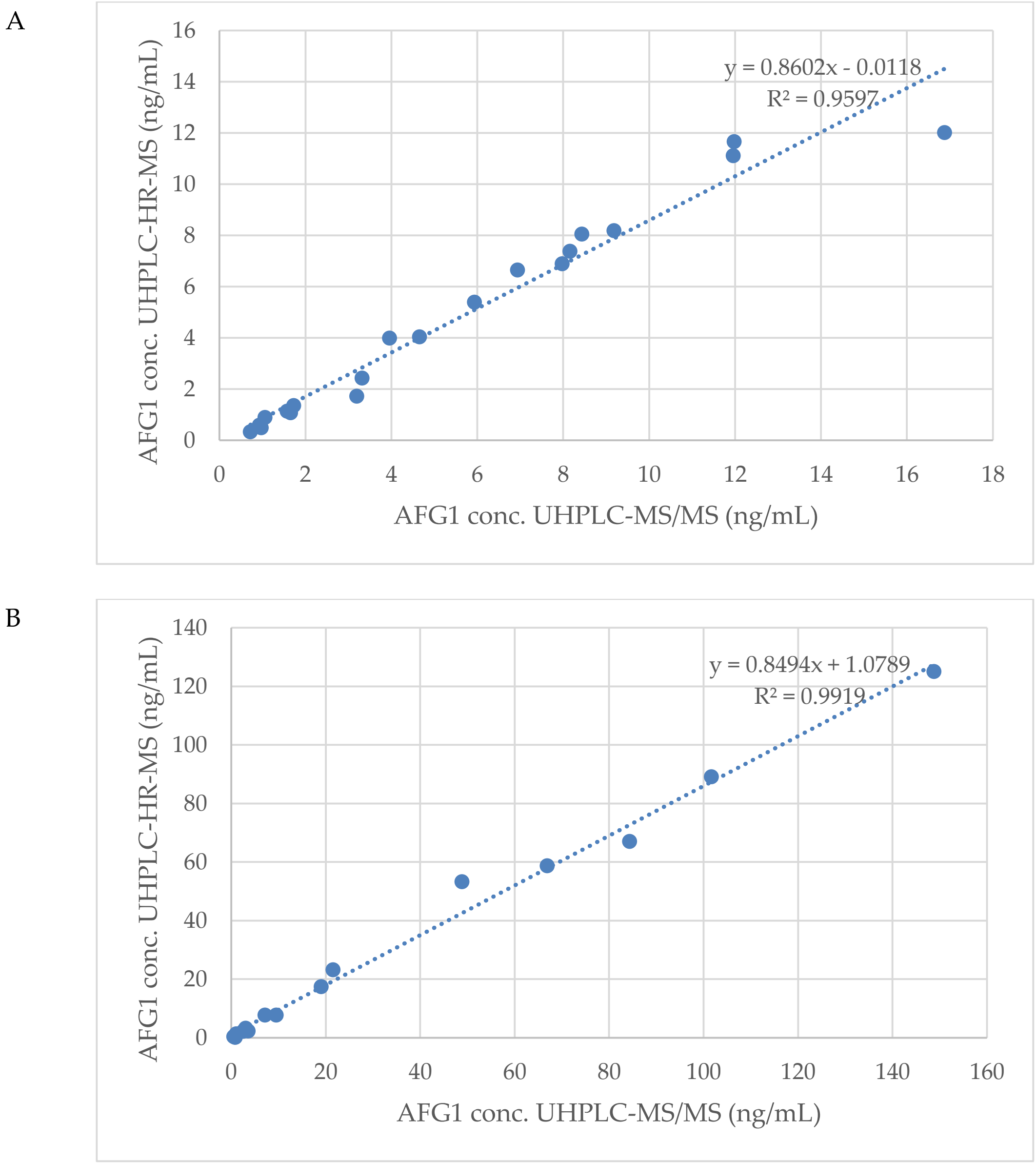

Figure S4. Correlation between the AFG1 plasma concentrations in 2 chickens that received an (A) oral and (B) intravenous administration of 2 mg/kg BW, after quantitative analysis using the UHPLC-MS/MS and UPLC-HRMS technique, respectively.
